# An application of fuzzy bipolar weighted correlation coefficient in decision-making problem

**DOI:** 10.1371/journal.pone.0283516

**Published:** 2023-12-19

**Authors:** Saima Mustafa, Sadia Mahmood, Zabidin Salleh

**Affiliations:** 1 Departemnt of Mathematics and Statistics, PMAS-Arid Agriculture University Rawalpindi, 46300, Pakistan; 2 Department of Mathematics, Faculty of Ocean Engineering Technology and Informatics, University Malaysia Terengganu, 21030 Kuala Nerus, Terengganu, Malaysia; UPSI: Universiti Pendidikan Sultan Idris, MALAYSIA

## Abstract

Diagnosing and finding the disease in medical sciences is a complex procedure. The basic steps involved in finding starts with signs, symptoms, and test. This study is based on the diagnosis of a skin disorder. The identification of a disease has been made on the basis of symptoms that sometimes show bipolarity. To address this bipolarity, the bipolar fuzzy sets are used as bipolar fuzzy sets cover the positive as well as negative aspects of a specific symptom. It is combined with the idea of soft sets, which gives more precise results. We have proposed a new technique in which a correlation coefficient is used to measure bipolar fuzzy soft set, which has been applied for diagnosis. The BFSSs deal most effectively with dual and fuzzy information. The correlation coefficient and the weighted correlation coefficient of BFSSs are suggested in this research. Based on said techniques, the decision-making method is suggested under a bipolar fuzzy environment to resolve ambiguous and unclear information. The implementation and effectiveness of the proposed and existing strategy has been checked by numerical computation.

## 1 Introduction

Decision making plays an important role in every aspect of life. In this study, we have combined the decision making concept with weighted fuzzy bipolar correlation coefficient. Correlation plays an important role in different disciplines named as Statistics, engineering, and social sciences. This is used to assess the interdependence of two variables based on their reciprocal relationship. This is very important concept not in statistics but also in probabilistic theory. The probabilistic techniques can be utilized to tackle a wide range of real-world engineering issues, probability strategy has some limitations; for example, the process’s probability is determined by a significant amount of random data. On the other hand, large complex systems have many ambiguities, making it challenging to obtain precise probability occurrences. As a result, probability theory outcomes do not always provide meaningful information for experts due to a lack of quantitative information/data. Results based on probability theory are not always available to the general public due to the earlier challenges. As a result, probabilistic approaches are frequently insufficient to resolve such intrinsic data uncertainties. A lot of studies around the world have addressed and suggested various techniques to solving problems with uncertainty. To initiate, (Zadeh 1965) in [1], devised the concept of a fuzzy set to handle problems involving ambiguity and uncertainty. (Yu, 1993), in [2], introduced the correlation of fuzzy numbers. (Chiang & Lin, 1999), in [3], studied the fuzzy correlation of imprecise information by using the following strategies for typical statistics. As a result, they introduced the concept of the flexible correlation coefficient, which enlightens the fuzzy set.

It has been observed that many developments of new theories have been made, but they have limitations due to their insufficient parameterization tool. As a result, the decision-maker(s) cannot make an appropriate decision due to uncertain information.

The concept of fuzzy set theory was then polished by (Molodtsov, 1999) in [4], introduced the concept of soft set (SS) theory, which assigns ratings to parameters.to overcome these benefits. In [5, 6,7], Chen, C.-T. (2000), Chen, T.-Y., & Tsao, C.-Y. (2008) and (Maji et al., 2002,), developed, group topsis concept, fuzzy soft set (FSS) and intuitionistic fuzzy soft set (IFSS) as extensions of this theory by combining existing FS and IFS theoretical approaches. But it is experienced that in some circumstances, we must thoroughly examine both the positive and negative aspects of the problem, which can’t be addressed with fuzzy sets. To address these issues, (W.-R. Zhang, 1998) in [8], originally presented bipolar fuzzy sets as a fuzzy set extension. The concept of bipolarity is fundamental in separating positive and negative information.

The positive information in bipolarity represents the level of satisfaction, while the negative information shows the level of unhappiness. In BFSS theory, expert preferences are expressed as a set of parameters. For instance, in case an individual needs to buy a mobile, then according to the BFS theory, the information is captured corresponding to a single parameter on two classification- one is satisfaction degree. The other is the degree of dissatisfaction, whereas, in BFSS, deep insight about the mobile specification is considered such as ’price,’ ’camera, ’RAM,’ ’screen size,’ etc. In [ 9,10, 11, 12, 13], (M. Sarwar & Akram, 2017; Musavarah Sarwar & Akram, 2017, Akram et.al 2019 and Akram et. al 2020, Akram et. al 2020 ), investigated some applications on bipolar fuzzy sets. Despite all of these possibilities of bipolar fuzzy sets, it is still important to incorporate all of the data into a valid decision-making model and analyze it in an organized manner to maintain an acceptable knowledge representation framework. As a result, in [14], (Alghamdi et al., 2018), proposed bipolar fuzzy multi-criteria decision-making models that evaluated options for several criteria using bipolar fuzzy values. Moreover, Shimaiza et al. (2019), see [15, 16], explored some concepts on multicriteria decision making and bipolar fuzzy sets. Furthermore, bipolarity and fuzziness are two distinct but overlapping concepts used to describe various characteristics of the human mind. While dealing with ambiguity, a decision-maker concentrates on language imprecision.

Decision-making is a procedure of deciding or classifying an alternative from a set of available options. The most important factors are the source of information, collection of alternatives, and preference values in which decision is made. Technique for Order Preference by Similarity to Ideal Solution (TOPSIS) is a famous approach to deal with Multi-Criteria Decision-Making Method that was first proposed by (Hwang & Yoon, 1981) see [17], expanded the conventional TOPSIS to Fuzzy TOPSIS and addressed a fuzzy information decision-making problem. Each criterion’s weight and rating of each possibility are described in linguistic terms in his work. Since then, the extended fuzzy TOPSIS technique has expanded its scope to include many applications, including logistics, human resources, and management. (Li & Nan, 2011), in [18], proposed the TOPSIS approach in an intuitionistic fuzzy framework and was also addressed by (Joshi & Kumar, 2014), see [19]. (S. P. Wan et al., 2015;in [20] and Shuping Wan et al., 2016, [21]) introduced a novel method using interval-valued Atanassov intuitionistic fuzzy preference relation for group decision making. (Dong & Wan, 2016), [22], apply TOPSIS technique for virtual enterprise partner selection. Further, (Dey et al., 2016),[23] approach for handling decision-making problems in a bipolar neurosophic environment. After this formation, numerous scholars have applied the TOPSIS Method to decision-making in fuzzy intuitionistic fuzzy, inter-valued fuzzy, and hypersoft sets environments. (Shuping Wan & Dong, 2020) [24], proposed a method for Atanassov’s Interval-Valued Intuitionistic Fuzzy MAGDM with Incomplete Attribute Weight Information For the extended TOPSIS method based on correlation coefficient, the reader is referred to (Garg & Arora, 2020), see [25] , (Lin et al., 2019) in [26] , (R. M. Zulqarnain et al., 2020; Rana Muhammad Zulqarnain et al., 2021), see [27, 28].

Although the approaches described above are quite helpful in handling many decision-making difficulties, they do have certain drawbacks:

1. The weights of each Decision Maker in the Decision-Making (DM) technique are frequently seen as the same concerning diverse qualities, which is quite illogical and impractical. Every DM is good at only one subject in a real-world decision situation. As a result, allocating various weights to different features for each decision-maker is more natural and reasonable.
2. The multicriteria decision making problem is solved using the BF -TOPSIS approach. The data structure is unique in that no preference is taken into account while selecting alternatives.
3. The distance measure was employed in the topsis procedure to obtain optimal answers. However, the topsis approach based on distance cannot always determine the optimal solution, it is necessary to improve the BF TOPSIS method by using other measurements such as correlation coefficient.

We suggested a new approach based on correlation co-efficient to overcome these flaws in a bipolar fuzzy environment. It has been mentioned here that Chiang, D.-A., & Lin, N. P. (1999), in [3], described the coorrelation coefficient with respect to fuzzy sets. Here we have used bipolar concept for the coorrelation coefficient. Our contribution can be summarized as:

1. Based on positive and negative membership degrees, we developed a connection between correlation coefficient and a weighted correlation coefficient for bipolar fuzzy soft sets and then examined their features.
2. Different weights are allocated for each decision-maker concerning different attributes or criteria, which is natural in real-world decision-making.
3. Bipolar fuzzy soft sets are introduced by merging bipolar fuzzy sets with soft sets to produce an order of preferences that leads to more exact outcomes.
4. The proposed correlation co-efficient is used to modify the TOPSIS method for bipolar fuzzy soft sets.

The rest of the research is structured as follows. In section one, some definitions such as Fuzzy Set, Soft set, Fuzzy Soft set, Bipolar Fuzzy set, and Bipolar Fuzzy Soft set have been recalled, which have been used to construct the structure of further work. In section two, the correlation coefficient and informational energies of Bipolar Fuzzy Soft sets are suggested, and the correlation coefficient and the weighted correlation coefficient of Bipolar Fuzzy Soft sets are developed. In the third section, the properties are proved by utilizing the correlation coefficient and informational energies. A modified technique is suggested under a Bipolar Fuzzy environment to resolve confusing and unclear problems. A diagnosis analysis example is given in the fourth section to check the applicability of the proposed technique. Finally, comparative analysis has been organized.

## 2. Preliminaries

In this section, we will go over some basic definitions like fuzzy set (FS), soft sets (SSs), fuzzy soft sets (FSSs), bipolar fuzzy set (BFS), and bipolar fuzzy soft sets (BFSSs). These definitions have been used to construct the structure of further work and for better understanding.

**Definition 3.1:** A fuzzy set *χ* in the universe of information *U* can be demonstrated as a set of ordered pairs, and it can be represented mathematically as

$\chi =\{(y,\mu_{x}(y))|y\in U\}$, where *χ* degree of membership of *y* that assumes values in the range from 0 to 1, i.e., *μ*_*x*_(*y*)∈[0,1].

**Definition 3.2:** Let *U* be the universal set and ∈ be the set of attributes concerning *U*. Let *P*(*U*) be the power of *U* and *A*⊆∈ then, a pair (*F*,*A*) is called soft sets over *U* where F is a mapping from *A* to *P*(*U*), i.e., as *F*: *A*→*P*(*U*). It is defined as (*F*,*A*) = *F*(*e*)∈*P*(*U*).

**Definition 3.3:** Let *A*,*B*⊂*ε*, where *ε* be a set of parameters and (*F*,*A*), (*G*,*B*), be two Soft sets over U. Then, the basic operations over them are indicated as:

1. (*F*,*A*)⊆(*G*,*B*) if *A*⊆*B* and *F*(*e*)⊆*G*(*e*)∀*e*∈*A*.
2. If (*F*,*A*)⊆(*G*,*B*) and (*G*,*B*)⊆(*F*,*A*).
3. Complement (*F*,*A*)^*c*^ = (*F*^*c*^,*A*) where *F*^*c*^: *A*→*K*^*U*^ defined as *F*^*c*^(*e*) = *U*−*F*(*e*)∀*e*∈*A*, where *K*^*U*^ is a set of all subsets of U.

**Definition 3.4:** A bipolar fuzzy set *A* in a universe *U* is as an object having the form:

$$A=\{(\mu ,(\lambda_{A}^{+}(u),\lambda_{A}^{-}(u))|u\in U\},$$

where, $\lambda_{A}^{+}(u):u\to [0,1]$, $\lambda_{A}^{+}(u):u\to [-1,0]$, so $\lambda_{A}^{+}(u)$ denotes the positive information (satisfaction degree) and $\lambda_{A}^{-}(u)$ denotes the negative information (dis-satisfaction degree).

**Definition 3.5:** A mapping *F*: *A*→*BF*^*u*^*F*: *A*→*BF*^*u*^ is called as bipolar fuzzy soft sets defined as

$$F_{u_{i}}(e_{j}))=\{u_{i},(\lambda_{j}^{+}(u),\lambda_{j}^{-}(u)|u_{i}\in U)$$

where *BF*^*u*^ is the bipolar fuzzy soft sets of *U* and $\lambda_{j}^{+}$ and $\lambda_{j}^{-}$ are satisfaction and dissatisfaction degrees, respectively? For simplicity, we denoted the pair $F_{u_{i}}(e_{j}))$ as $\alpha_{ij}=(\lambda_{ij}^{+},\lambda_{ij}^{-})$ or $\left(F,A)=(\lambda_{ij}^{+},\lambda_{ij}^{-}\right)$ and called as a bipolar fuzzy soft number.

### Motivation

Dual aspects of bipolar critical thinking on positive and negative aspects are used in a wide variety of decision-making. For example, in decision and coordination, the two sides are frequently the effects and side effects, like and unlike, cooperation and competition. In this case, the decision analyst will need to know about the notion of bipolar fuzzy sets. The bipolar fuzzy sets (BFS) are another modification of the fuzzy set (FS) by adding a negative membership function. To choose the optimal option, various decision approaches are utilized, one of which is the technique for order preference by similarity to the ideal solution, named as Topsis Therefore Further it was extended from traditional TOPSIS to fuzzy TOPSIS and then into bipolar topsis, (BF- TOPSIS). The said method has been applied to solve the medical-related problem (medical diagnosis). Our main goal for writing this research is to offer a new correlation coefficient for BFSSs data and build the TOPSIS method for BFSSs using the proposed correlation coefficient. We presented a new BFSS correlation coefficient and investigated various aspects of the generated correlation coefficient to quantify the dependency on BFSSs. We created an algorithm to solve MADM issues using the extended TOPSIS approach, and checked the validity of the proposed technique with a numerical illustration. To find similarity measures and distance, the general closeness coefficient is employed. Meanwhile, the correlation coefficient is employed to calculate the closeness coefficient in our suggested method.

Proposed Approach

## 3. Correlation coefficients for BFSSs

Let *U* = {*u*_1_, *u*_2_,….*u*_*n*_} be a set of professionals and *ε* = {*e*_1_, *e*_2_,….*e*_*m*_} be the set of parameters. In this section, we suggest some correlation coefficients under the bipolar fuzzy environment, which are defined as

**Definition 3.1:** Let $\left(G,\varepsilon )=\{u_{i},\lambda_{G_{j}}^{+}(u_{i}),\lambda_{G_{j}}^{-}(u_{i})|u_{i}\in U\right\}$ and $\left(H,\varepsilon )=\{u_{i},\lambda_{H_{j}}^{+}(u_{i}),\lambda_{H_{j}}^{-}(u_{i})|u_{i}\in U\right\}$ be two BFSSs defined over a set of parameters *ε* = {*e*_1_, *e*_2_,….*e*_*m*_}. Then the informational energies of two BFSSs (*G*, *ε*) and (*H*, *ε*) are expressed as;

$$I_{BFSS}(G,\varepsilon )=\sum_{j=1}^{m}\sum_{i=1}^{n}[(\lambda_{G_{j}}^{+}(u_{i}){]}^{2}+[(\lambda_{G_{j}}^{-}(u_{i}){]}^{2},$$ (3.1)

$$I_{BFSS}(H,\varepsilon )=\overset{m}{\underset{j=1}{\sum }}\overset{n}{\underset{i=1}{\sum }}[{\left(\lambda_{H_{j}}^{+}(u_{i})\right]}^{2}+[{\left(\lambda_{H_{j}}^{-}(u_{i})\right]}^{2}$$ (3.2)

The correlation of two BFSSs (*G*, *ε*), and (*H*, *ε*) can be expressed as

$$C_{BFSS}((G,\varepsilon ),(H,\varepsilon ))=\sum_{j=1}^{m}\sum_{i=1}^{n}[(\lambda_{G_{j}}^{+}(u_{i})\cdot \lambda_{H_{j}}^{+}(u_{i})+{\left(\lambda_{G_{j}}^{-}(u_{i})..\lambda_{H_{j}}^{-}(u_{i})\right]}^{2}$$ (3.3)

In Eq (3.1) expressions $\lambda_{G_{j}}^{+}(u_{i})$ represents the positive membership function and $\lambda_{G_{j}}^{-}(u_{i})$ represents negative membership function of the BFSS (*G*, *ε*), where as in Eq (3.2), $\lambda_{H_{j}}^{+}(u_{i})$ and $\lambda_{H_{j}}^{-}(u_{i})$ shows the positive and negative membership function of the BFSS (*H*, *ε*) respectively. The correlation between both (*G*, *ε*) and (*H*, *ε*)) bipolar fuzzy soft sets are expressed in Eq (3.3).

The following proposition established the relationship between correlation of bipolar fuzzy soft set and their informational energies defined over the parameters

**Proposition1:**Let (*G*, *ε*) = $\left\{u_{i},((\lambda_{G_{j}}^{+}(u_{i}).((\lambda_{G_{j}}^{-}(u_{i})|u_{i}\in U\right\}$ and $\left(H,\in )=\{u_{i},((\lambda_{H_{j}}^{+}(u_{i}).((\lambda_{H_{j}}^{-}(u_{i})|u_{i}\in U\right\}$ be two BFSSs and *C*_*BFss*_((*G*, ∈), (*H*, *ε*)) be the correlation between them, then following properties holds:

1. *C*_*BFss*_((*G*, ∈), (*G*, *ε*)) = *I*_*BFSS*_(*G*, ∈).
2. *C*_*BFss*_((*G*, ∈), (*H*, *ε*)) = *C*_*BFSS*_(*H*, ∈), (*G*, ∈)).

**Definition 3.2:** The correlation coefficient between two BFSSs (*G*, *ε*) and (*H*, *ε*) is *ρ*1((*G*, *ε*), (*H*, *ε*) and is described as:

$$\rho_{1}((G,\varepsilon ),(H,\varepsilon )=\frac{C_{BFSS}((G,\in ),(H,\in ))}{\sqrt{I_{BFSS}((G,\in )..I_{BFSS}(H,\in )}}$$

$$=\frac{C_{BFSS}((G,\in ),(H,\in )}{\sqrt{I_{BFSS}((G,\in )..I_{BFSS}(H,\in )}}$$

$$==\frac{\sum_{j=1}^{m}\sum_{i=1}^{n}\left(\lambda_{G_{j}}^{+}(u_{i}),\lambda_{H_{j}}^{+}(u_{i})+\lambda_{G_{j}}^{-}(u_{i}),\lambda_{H_{j}}^{-}(u_{i})\right)}{\sqrt{\sum_{j=1}^{m}\sum_{i=1}^{n}(\lambda_{G_{j}}^{+2}(u_{i})+\lambda_{G_{j}}^{-2}(u_{i})}.\sqrt{\sum_{j=1}^{m}\sum_{i=1}^{n}(\lambda_{H_{j}}^{+2}(u_{i})+\lambda_{H_{j}}^{-2}(u_{i})}}$$ (3.4)

The correlation coefficient ρ_1_ described in Eq (3.4) holds the properties of correlation coefficient which is shown in the proposition below

**Proposition 2:** The correlation coefficient between two BFSS (*G*, *ε*) and (*H*, *ε*) stated in Eq (3.4) holds the following properties:

1. 0≤*ρ*_1_((*G*, ∈), (*H*, ∈))≤1;
2. *ρ*_1_((*G*, ∈), (*H*, ∈)) = *ρ*_1_((*H*, ∈), (*G*, ∈));
3. If (*G*, *ε*) = (*H*, *ε*) i.e. if for all i, j, $\lambda^{+}{}_{G_{j}}(u_{i})=\lambda_{H_{j}}^{+}(u_{i})$, and $\lambda^{-}{}_{G_{j}}(u_{i})=\lambda^{-}{}_{H_{j}}(u_{i})$, then *ρ*_1_((*G*, ∈), (*H*, ∈)) = 1.

***(Property 1)*:** 0≤*ρ*_1_((*G*, *ε*), (*H*, ∈))≤1;

Proof:

Consider the property 1, the inequality *ρ*_1_((*G*, *ε*), (*H*, ∈))≥0 ≥ is obvious and here we only have to prove *ρ*_1_((*G*, *ε*), (*H*, ∈))≤1.


$$C_{BFSS}((G,\varepsilon ),(H,\in ))=\sum_{j=1}^{m}\sum_{i=1}^{n}[(\lambda_{G_{j}}^{+}(u_{i}).(\lambda_{H_{j}}^{+}(u_{i})+(\lambda_{G_{j}}^{-}(u_{i}).(\lambda_{H_{j}}^{-}(u_{i})].$$

By expanding the summation of the above expression where (*i* = 1,2,3…n)

$$=\left[\begin{array}{l} \sum_{j=1}^{m}[\lambda_{G_{j}}^{+}(u_{1}).(\lambda_{H_{j}}^{+}(u_{1})+(\lambda_{G_{j}}^{-}(u_{1}).(\lambda_{H_{j}}^{-}(u_{1})]+\\ \sum_{j=1}^{m}[\lambda_{G_{j}}^{+}(u_{2}).(\lambda_{H_{j}}^{+}(u_{2})+(\lambda_{G_{j}}^{-}(u_{2}).(\lambda_{H_{j}}^{-}(u_{2})]+\\ \sum_{j=1}^{m}\left[\lambda_{G_{j}}^{+}(u_{n}).(\lambda_{H_{j}}^{+}(u_{n})+(\lambda_{G_{j}}^{-}(u_{n}).(\lambda_{H_{j}}^{-}(u_{n})\right] \end{array}\right]$$

After expanding the second summation where (*j* = 1,2,3…m), we get

$$=\left[\begin{array}{l} \sum_{j=1}^{m}[\lambda_{G_{j}}^{+}(u_{1}).(\lambda_{H_{j}}^{+}(u_{1})+(\lambda_{G_{j}}^{-}(u_{1}).(\lambda_{H_{j}}^{-}(u_{1})]+\\ \sum_{j=1}^{m}[\lambda_{G_{j}}^{+}(u_{2}).(\lambda_{H_{j}}^{+}(u_{2})+(\lambda_{G_{j}}^{-}(u_{2}).(\lambda_{H_{j}}^{-}(u_{2})]+\\ \sum_{j=1}^{m}\left[\lambda_{G_{j}}^{+}(u_{n}).(\lambda_{H_{j}}^{+}(u_{n})+(\lambda_{G_{j}}^{-}(u_{n}).(\lambda_{H_{j}}^{-}(u_{n})\right] \end{array}\right]$$

$$=[\begin{array}{l} (\lambda_{G_{1}}^{+}(u_{1}).(\lambda_{H_{1}}^{+}(u_{1})+(\lambda_{G_{1}}^{-}(u_{1}).(\lambda_{H_{1}}^{-}(u_{1})+(\lambda_{G_{2}}^{+}(u_{1}).(\lambda_{H_{2}}^{+}(u_{1})+(\lambda_{{G_{1}}_{2}}^{-}(u_{2}).(\lambda_{H_{2}}^{-}(u_{1})+\\ \left(\lambda_{G_{m}}^{+}(u_{1}).(\lambda_{H_{m}}^{+}(u_{1})+(\lambda_{G_{m}}^{-}(u_{1}).(\lambda_{H_{m}}^{-}(u_{1})\right) \end{array}]+$$

$$[\lambda_{G_{1}}^{+}(u_{2}).(\lambda_{H_{1}}^{+}(u_{2})+(\lambda_{G_{1}}^{-}(u_{2}).(\lambda_{H_{1}}^{-}(u_{2}))+(\lambda_{G_{2}}^{+}(u_{2}).(\lambda_{H_{2}}^{+}(u_{2})+(\lambda_{{G_{1}}_{2}}^{-}(u_{2}).(\lambda_{H_{2}}^{-}(u_{2})+\ldots \ldots \ldots \ldots \ldots \ldots \ldots \ldots \ldots \ldots \ldots ..$$

$$+(\lambda_{G_{m}}^{+}(u_{2}).(\lambda_{H_{m}}^{+}(u_{2})+(\lambda_{G_{m}}^{-}(u_{2}).(\lambda_{H_{m}}^{-}(u_{2})]+------+[\lambda_{G_{1}}^{+}(u_{n}).(\lambda_{H_{1}}^{+}(u_{n})+(\lambda_{G_{1}}^{-}(u_{n}).(\lambda_{H_{1}}^{-}(u_{n})]+$$

$$[(\lambda_{G_{2}}^{+}(u_{n}).(\lambda_{H_{2}}^{+}(u_{n})+(\lambda_{{G_{1}}_{2}}^{-}(u_{2}).(\lambda_{H_{2}}^{-}(u_{2})]+------+[(\lambda_{G_{m}}^{+}(u_{n}).(\lambda_{H_{m}}^{+}(u_{n})+(\lambda_{G_{1m}}^{-}(u_{2}).(\lambda_{H_{m}}^{-}(u_{n})].$$

After summating the above whole expression, we get the following

$$=\sum_{j=1}^{m}\left[(\lambda_{Gj}^{+}(u_{1}).(\lambda_{H_{j}}^{+}(u_{1}\right)+(\lambda_{Gj}^{+}(u_{2}).(\lambda_{H_{j}}^{+}(u_{2}))+\ldots .+(\lambda_{Gj}^{+}(u_{n}).(\lambda_{H_{j}}^{+}(u_{n}))]+\sum_{j=1}^{m}\left[(\lambda_{Gj}^{-}(u_{1}).(\lambda_{H_{j}}^{-}(u_{1}\right)+(\lambda_{Gj}^{-}(u_{2}).(\lambda_{H_{j}}^{-}(u_{2}))+\ldots .+]\lambda_{Gj}^{-}(u_{n}).(\lambda_{H_{j}}^{-}(u_{n}))].$$

By applying Cauchy- Schwarz inequality on above equation, we proceed by using the concept of correlation coefficient

$$=((a_{1}b_{1}+a_{2}b_{2}+\ldots .a_{n}b_{n}{)}^{2}\leq (a_{1}^{2}+a_{2}^{2}+\ldots .+a_{n}^{2})(b_{1}^{2}+b_{2}^{2}+\ldots .+b_{n}^{2})$$

We have $\left(C_{BFSS}((G,\varepsilon ),(H,\varepsilon ){)}^{2}\leq [[\sum_{j=1}^{m}(\lambda_{G_{j}}^{+2}(u_{1})+\lambda_{G_{j}}^{+2}(u_{2})+\ldots ..+\lambda_{G_{j}}^{+2}(u_{2})]+[\sum_{j=1}^{m}\left(\lambda_{G_{j}}^{-2}(u_{1})+\lambda_{G_{j}}^{-2}(u_{2})+\ldots ..+\lambda_{G_{j}}^{+2}(u_{2})\right]\right]$ $*[\sum_{j=1}^{m}(\lambda_{H_{j}}^{+2}(u_{1})+\lambda_{H_{j}}^{+2}(u_{2})+\ldots ..+\lambda_{H_{j}}^{+2}(u_{2})]+[\sum_{j=1}^{m}\left(\lambda_{H_{j}}^{-2}(u_{1})+\lambda_{H_{j}}^{-2}(u_{2})+\ldots ..+\lambda_{H_{j}}^{+2}(u_{2})\right]$

$$=\left\{\sum_{j=1}^{m}\sum_{i=1}^{n}\left(\lambda_{G_{j}}^{+2}(u_{i})+\lambda_{G_{j}}^{-2}(u_{i}\right)\right\}\times \left\{\sum_{j=1}^{m}\sum_{i=1}^{n}\left(\lambda_{H_{j}}^{+2}(u_{i})+\lambda_{H_{j}}^{-2}(u_{i}\right)\right\}$$

where, $I_{BFSS}(G,\varepsilon )=\{\sum_{j=1}^{m}\sum_{i=1}^{n}\left(\lambda_{G_{j}}^{+2}(u_{i})+\lambda_{G_{j}}^{-2}(u_{i}\right)\}$

$$I_{BFSS}(H,\varepsilon )=\left\{\sum_{j=1}^{m}\sum_{i=1}^{n}\left(\lambda_{H_{j}}^{+2}(u_{i})+\lambda_{H_{j}}^{-2}(u_{i}\right)\right\}$$

$$=I_{BFSS}(G,\varepsilon ).I_{BFSS}(H,\varepsilon ).$$

From this we deduce the following:

Therefore, $(C_{BFSS}((G,\varepsilon ),(H,\varepsilon ){)}^{2}\leq I_{\mathrm{BFSS}}(G,\in ).I_{\mathrm{BFSS}}(H,\in ).$ Hence, by using Eq (3.4), we get *ρ*_1_((*G*, ∈), (*H*, ∈))≤1 and 0≤*ρ*_1_((*G*, ∈), (*H*, ∈))≤1.

***(Property 2)*** *ρ*_1_((*G*, ∈), (*H*, ∈)) = *ρ*_1_((*H*, ∈), (*G*, ∈)).

Proof:

$$\rho_{1}(\left(G,\in ),(H,\in \right))=\frac{\sum_{j=1}^{m}\sum_{i=1}^{n}\left(\lambda_{G_{j}}^{-}(u_{i}).\lambda_{H_{j}}^{-}(u_{i})+\lambda_{G_{j}}^{+}(u_{i}).\lambda_{H_{j}}^{+}(u_{i}\right)}{\sqrt{\sum_{j=1}^{m}\sum_{i=1}^{n}\lambda_{H_{j}}^{+2}(u_{i})+\lambda_{H_{j}}^{-2}(u_{i})}.\sqrt{\sum_{j=1}^{m}\sum_{i=1}^{n}\lambda_{G_{j}}^{+2}(u_{i})+\lambda_{G_{j}}^{-2}(u_{i})}}$$

$$=\frac{\sum_{j=1}^{m}\sum_{i=1}^{n}\left(\lambda_{G_{j}}^{+}(u_{i}).\lambda_{H_{j}}^{+}(u_{i})+\lambda_{G_{j}}^{-}(u_{i}).\lambda_{H_{j}}^{-}(u_{i}\right)}{\sqrt{\sum_{j=1}^{m}\sum_{i=1}^{n}\lambda_{G_{j}}^{+2}(u_{i})+\lambda_{G_{j}}^{-2}(u_{i})}.\sqrt{\sum_{j=1}^{m}\sum_{i=1}^{n}\lambda_{H_{j}}^{+2}(u_{i})+\lambda_{H_{j}}^{-2}(u_{i})}}$$

$$=\rho_{1}(\left(H,\in ),(g,\in \right)).$$

Hence proved that *ρ*_1_((*G*, ∈), (*H*, ∈)) = ***ρ***_**1**_((***H***, ∈), (***g***, ∈)).

***(Property 3)*:** If (*G*, ∈) = (*H*, ∈) i.e. if ∀ i, j, $\lambda_{G_{j}}^{+}(u_{i})=\lambda_{H_{j}}^{+}(u_{i})$ and $\lambda_{G_{j}}^{-}(u_{i})=\lambda_{H_{j}}^{-}(u_{i})$, then ***ρ***_**1**_((***H***, ∈), (***g***, ∈)) = **1.**

Proof:

As we have $\lambda_{G_{j}}^{+}(u_{i})=\lambda_{H_{j}}^{+}(u_{i})$ and $\lambda_{G_{j}}^{-}(u_{i})=\lambda_{H_{j}}^{-}(u_{i})$, ∀ i, j, then from Eq (3.4), we get

$$\rho_{1}(\left(G,\in ),(H,\in \right))=\frac{C_{BFSS}((G,\varepsilon )(H,\in ))}{\sqrt{I_{BFSS}(G,\varepsilon )I_{BFSS}(H,\varepsilon )}}$$

$$=\frac{C_{BFSS}((G,\varepsilon )(H,\in ))}{\sqrt{I_{BFSS}(G,\varepsilon )I_{BFSS}(H,\varepsilon )}}$$

$$=\frac{\sum_{j=1}^{m}\sum_{i=1}^{n}\left(\lambda_{H_{j}}^{+}(u_{i})).\lambda_{H_{j}}^{+}(u_{i})+\lambda_{H_{j}}^{-}(u_{i}).\lambda_{H_{j}}^{-}(u_{i}\right)}{\sqrt{\sum_{j=1}^{m}\sum_{i=1}^{n}\left(\lambda_{H_{j}}^{+2}(u_{i})+\lambda_{H_{j}}^{-2}(u_{i}\right)}\sqrt{\sum_{j=1}^{m}\sum_{i=1}^{n}\left(\lambda_{H_{j}}^{+2}(u_{i}))+(\lambda_{H_{j}}^{-2}(u_{i})\right)}}$$

$$=\frac{\sum_{j=1}^{m}\sum_{i=1}^{n}\left(\lambda_{H_{j}}^{+2}(u_{i})+\lambda_{H_{j}}^{-2}(u_{i}\right)}{\sqrt{\sum_{j=1}^{m}\sum_{i=1}^{n}\left(\lambda_{H_{j}}^{+2}(u_{i})+\lambda_{H_{j}}^{-2}(u_{i}\right)}\sqrt{\sum_{j=1}^{m}\sum_{i=1}^{n}\left(\lambda_{H_{j}}^{+2}(u_{i})+\lambda_{H_{j}}^{-2}(u_{i}\right)}}=1$$

Thus, *ρ*_1_((*G*, *ε*), (*H*, *ε*)) = 1, is the required result.

**Definition 3.3:** The correlation coefficient *ρ*_2_((*G*, *ε*), (*H*, *ε*)) between BFSSs (G,∈) and (H,∈) is expressed as:

$$\rho_{2}((G,\varepsilon ),(H,\varepsilon ))=\frac{C_{BFSS}((G,\in ),(H,\in ))}{\max\{I_{BFSS}(G,\in ),I_{BFSS}(H,\in )\}}$$

$$=\frac{\sum_{j=1}^{m}\sum_{i=1}^{n}(\lambda_{G_{j}}^{+}(u_{i}).\lambda_{H_{j}}^{+}(u_{i})+\lambda_{G_{j}}^{-}(u_{i})\lambda_{G_{j}}^{-}(u_{i})}{\max\left\{\sum_{j=1}^{m}\sum_{i=1}^{n}(\lambda_{G_{j}}^{+2}(u_{i})+\lambda_{G_{j}}^{-2}(u_{i}),\sum_{j=1}^{m}\sum_{i=1}^{n}\left(\lambda_{H_{j}}^{+2}(u_{i})+\lambda_{H_{j}}^{-2}(u_{i}\right)\right\}}$$ (3.5)

**Proposition 3:** The correlation coefficient *ρ*_2_((*G*, *ε*), (*H*, *ε*)) between two BFSSs (*G*, *ε*) and (*H*, *ε*)) expressed in equation Eq (3.5) satisfy the following properties:

1. 0≤*ρ*_2_((*G*, *ε*), (*H*, *ε*))≤1
2. *ρ*_2_((*G*, *ε*), (*H*, *ε*)) = *ρ*_1_((*H*, *ε*), (*G*, *ε*)).
3. If (*G*, ∈) = (*H*, ∈) i.e., if ∀ i, j, ${\lambda^{+}}_{G_{j}}(u_{i})={\lambda^{+}}_{H_{j}}(u_{i})$ and ${\lambda^{-}}_{G_{j}}(u_{i})={\lambda^{-}}_{H_{j}}(u_{i})$, then *ρ*_2_((*G*, *ε*), (*H*, *ε*)) = 1.

***(Property 1)*:** 0≤*ρ*_2_((*G*, *ε*), (*H*, *ε*))≤1

**Proof:** Since ((*G*, ∈), (*H*, ∈)) are BFSSs therefore, the inequality *ρ*_2_((*G*, *ε*), (*H*, *ε*))≥0 is obvious, and here we only have to prove *ρ*_2_((*G*, *ε*), (*H*, *ε*))≤1 by applying Cauchy Schwarz inequality,

$$\sum_{i=1}^{n}a_{i}b_{i}\leq \sqrt{(\sum_{i=1}^{n}a_{i}^{2})(\sum_{i=1}^{n}b_{i}^{2}}$$ (3.6)

with equality if and only if the two vectors *a* = (*a*_1_, *a*_2_,…*a*_*n*_) and *b* = (*b*_1_, *b*_2_,…*b*_*n*_) are linearly dependent. Also, from Eq (3.6), we have

$$\sum_{i=1}^{n}a_{i}b_{i}\leq \sqrt{(\sum_{i=1}^{n}a_{i}^{2})(\sum_{i=1}^{n}b_{i}^{2}}\leq \sqrt{\max\{(\sum_{i=1}^{n}a_{i}^{2})(\sum_{i=1}^{n}b_{i}^{2}){\}}^{2}}=\max\{\sum_{i=1}^{n}a_{i}^{2}\sum_{i=1}^{n}b_{i}^{2}\}$$

and hence by Eq (3.5), we have 0≤*ρ*_2_((*G*, *ε*), (*H*, *ε*))≤1. The proof of remaining properties is similar to proposition 2.

However, in everyday practical applications, the considerations of weight are essential.

The weights determine every decision’s outcome the decision-maker assigns to each object in the universe of discourse. As a result, it’s vital to consider the weight before making a decision. The distinctive set can take diverse weights and, in this way, the weight vector for the experts *u*_*i*_ and parameters *e*_*j*_ are described as. Consider

$$\omega =(\omega_{1},\omega_{2},\omega_{3},\ldots \omega_{n}),(i=1,2,3\ldots n),$$

$$\Omega =(\Omega_{1},\Omega_{2},\Omega_{3},\ldots \Omega_{n}),(i=1,2,3\ldots m),$$

where *ω* represents the weight vector for the experts u_i_ such that *ω*_*i*_≥0 and ∑_*i* = 1_*ω*_*i*_ = 1, and *Ω* represents the weight vector of the parameters ∈_*j*_ such that *Ω*>0 and $\sum_{j=1}^{m}\left(\Omega_{j}=1\right)$.

Additionally, the correlation coefficient *ρ*_1_, *ρ*_2_ are extended to Weighted Correlation Coefficient for BFSSs which are as follows:

**Definition 3.4:** For two BFSSs (G,E) and (H,E), the weighted correlation coefficient is written as:

$$\rho_{3}((G,\varepsilon ),(H,\in ))=\frac{C_{WBFSS}((G,\varepsilon ),(H,\in ))}{\sqrt{I_{WBFSS}(G,\varepsilon ).I_{WBFSS}(H,\varepsilon )}}$$

$$=\frac{\sum_{j=1}^{n}\in_{j}(\sum_{i=1}^{n}w_{i}((\lambda_{G_{j}}^{+}(u_{i}),(\lambda_{H_{j}}^{+}(u_{i}))+(\lambda_{G_{j}}^{-}(u_{i}),(\lambda_{H_{j}}^{-}(u_{i})}{\sqrt{\sum_{j=1}^{m}\in_{j}(\sum_{i=1}^{n}w_{i}((\lambda_{G_{j}}^{+2}(u_{i})+(\lambda_{G_{j}}^{-2}(u_{i}))}\sqrt{\sum_{j=1}^{m}\in_{j}(\sum_{i=1}^{n}w_{i}((\lambda_{H_{j}}^{+2}(u_{i})+(\lambda_{H_{j}}^{-2}(u_{i}))}}$$ (3.7)

If we assume *ω*_*i*_ = 1 and ∈_*j*_ = 1 in Eq (3.7), then the correlation coefficient *ρ*_3_((*G*, *ε*), (*H*, ∈)) reduces to *ρ*_1_((*G*, *ε*), (*H*, ∈)), defines in Eq (3.4)

**Proposition 4:** The correlation coefficient *ρ*_3_((*G*, *ε*), (*H*, ∈)) for two BFSSs (*G*, *ε*) and (*H*, ∈), fulfill the properties as follows:

1. 0≤*ρ*_3_((*G*, *ε*), (*H*, ∈))≤1.
2. *ρ*_3_((*G*, *ε*), (*H*, ∈)) = *ρ*_3_((*H*, *ε*), (*G*, ∈)).
3. If (*G*, *ε*) = (*H*, *ε*) i.e., if ∀ i, j, $\left(\lambda_{G_{j}}^{+}(u_{i})=\lambda_{H_{j}}^{+}(u_{i}\right)$ and $\left(\lambda_{G_{j}}^{-}(u_{i})=\lambda_{H_{j}}^{-}(u_{i}\right)$, then *ρ*_3_((*G*, *ε*), (*H*, ∈)) = 1.

**The proof** is the same as proposition 2.

**Definition 3.5:** The weighted correlation coefficient *ρ*_4_((*G*, *ε*), (*H*, ∈)) for two BFSSs (*G*, *ε*) and (*H*, *ε*), is described as:

$$\rho_{4}(\left(G,\varepsilon ),(H,\in \right))=\frac{C_{WBFSS}((G,\in ),(H,\in ))}{\max\{I_{WBSS}((G,\in ),I_{WBSS}(H,\varepsilon )\}}$$

$$=\sum_{j=1}^{m}\frac{\varepsilon_{j}(\sum_{i=1}^{n}w_{i}((\lambda_{G_{j}}^{+}(u_{i}).(\lambda_{H_{j}}^{+}(u_{i}))+(\lambda_{G_{j}}^{-}(u_{i}).(\lambda_{H_{j}}^{-}(u_{i})}{\max\{\sum_{j=1}^{m}\varepsilon_{j}(\sum_{i=1}^{n}w_{i}\{(\lambda_{G_{j}}^{+2}(u_{i})+(\lambda_{H_{j}}^{-2}(u_{i})),\sum_{j=1}^{m}\in_{j}(\sum_{i=1}^{n}w_{i}(\lambda_{G_{j}}^{+2}(u_{i})+\lambda_{H_{j}}^{-2}(u_{i}))}$$ (3.8)

If we assume *w*_*i*_ = 1 and ∈_*j*_ = 1 in Eq (3.8), then the correlation coefficient *ρ*_4_((*G*, *ε*), (*H*, ∈)) reduces to *ρ*_2_((*G*, *ε*), (*H*, ∈)) defines in Eq (3.5)

**Proposition 5:** The correlation coefficient between two BFSS (*G*, *ε*) and (*H*, *ε*) indicated as *ρ*_4_((*G*, *ε*), (*H*, ∈)) expressed in Eq (3.8) satisfied the same properties as those in proposition 4.

**The proof** is similar to Proposition 3.

Using the above-defined correlation coefficient, we can quickly examine that correlation coefficient given in Eqs (3.4) and (3.7), utilizing the GM between the information energies of BFSSs (*G*, *ε*) and (*H*, *ε*).

Inspired by the paper as mentioned above (Akram et al., 2020), in the next section, we describe a method for solving decision-making difficulties based on the measure of correlation coefficient in a bipolar fuzzy environment. Here, we will propose a new algorithm for the bipolar fuzzy soft set, which is based on the correlation coefficient measure.

### Proposed algorithm for BFSSs based on the correlation coefficient

Step1: Define the problem by identifying the alternative and attributes.

Step 2: Construct a bipolar fuzzy decision matrix.

Step 3: Attributes (criteria) weights are assigned by the decision-maker.

Step 4: Compute the weighted bipolar decision matrix.

Step 5: Construct the correlation coefficient matrix.

Step 6: Compute indices to find PIA and NIA.

Step 7: Find correlation coefficient between weighted decision matrix of each alternative and PIA.

Step 8: Compute the closeness coefficient of each alternative.

Step 9: Rank the alternative by arranging the values in descending order.

Step 10: Select the best alternative.

Step 10: Select the best alternative.

**Fig 1:**
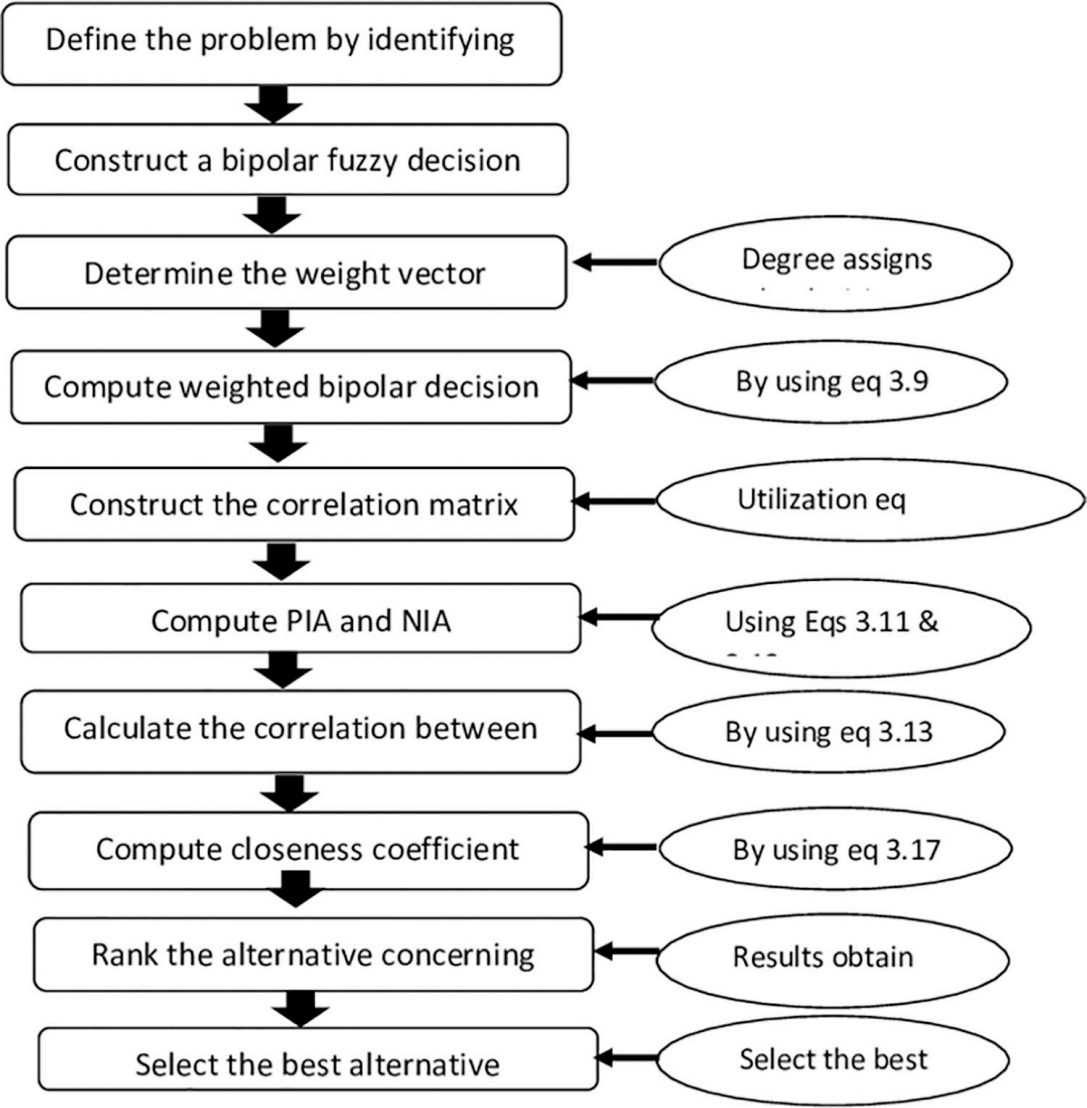
The step-wise resolution procedure of proposed algorithm with concerned equation.

Now, we validate the mathematical explanation of the algorithm mentioned in Fig 1.

### A mathematical explanation of the proposed methodology

**Step 1: Define the problem** (determination of alternatives and criteria)

Firstly, we define the set of decision maker, set of alternatives, and set of parameters which are given as

$$U_{i}=\{u_{1},u_{2},u_{3},\ldots u_{n}\},i=(1,2,3\ldots n)$$

$$\gamma^{2}=\{\gamma^{1},\gamma^{2},\gamma^{3},\ldots \gamma^{k}\},z=(1,2,3\ldots k)$$

$$\in_{j}=\{e_{1},e_{2},\ldots ..e_{m}\},j=(1,2,3\ldots m)$$

where, *u*_1_, *u*_2_, *u*_3_,…*u*_*n*_ represents the number of the decision-maker, *γ*^1^, *γ*^2^, *γ*^3^,…*γ*^*k*^ shows the set of alternatives and *e*_1_, *e*_2_,…..*e*_*m*_. Represents the set of parameters.

*w* = {*w*_1_, *w*_2_,…..*w*_*n*_}^*T*^ shows the weight vector, where weights are assigned to each decision-maker according to their expertise and knowledge satisfying the condition of normality, that is $\sum_{i=1}^{n}w_{i}=1$, where *w*_*i*_ is the weight for an ith decision-maker.

Set of k alternatives is taken for evaluation under the professionals with weights *w* = {*w*_1_, *w*_2_,…..*w*_*n*_}^*T*^, the judgment is given on the set of parameters having weights *Ω*_*j*_ = (*Ω*_1_, *Ω*_2_,…*Ω*_*m*_)^*T*^ such that *Ω*_*j*_>0 and $\sum_{j=1}^{m}\Omega_{j}=1$.

The evaluation of alternatives by the professionals, on the parameters, is given as BFSNs denoted by $\gamma_{ij}^{\left(z\right)}=(\lambda_{ij}^{+z},\lambda_{ij}^{-z})$ such that $0\leq \lambda_{ij}^{+}\leq 1$ and $-1\leq \lambda_{ij}^{-}\leq 0$. The following is a summary of the procedure for selecting the best alternatives:

Step two is to create a choice matrix.

**Step 2: Formation of decision matrix**

The above bipolar fuzzy information is summarized in terms of a matrix concerning professionals’ positive and negative aspects and considering parameters. The data relating to each alternative *γ*^(*z*)^, *z* = (1,2,3,…*k*) is shown in a matrix as follows:

$$(\gamma^{\left(z\right)},\in )=\left[\begin{array}{l} \left(\lambda_{11}^{+(z)},\lambda_{11}^{-(z)}),(\lambda_{12}^{+(z)},\lambda_{12}^{-(z)})\ldots (\lambda_{1n}^{+(z)},\lambda_{1m}^{-(z)}\right)\\ \left(\lambda_{21}^{+(z)},\lambda_{21}^{-(z)}),(\lambda_{22}^{+(z)},\lambda_{22}^{-(z)})\ldots (\lambda_{2n}^{+(z)},\lambda_{2m}^{-(z)}\right)\\ \ldots \ldots \ldots \ldots \ldots \ldots \ldots \ldots \ldots \ldots \ldots \ldots \ldots \ldots \ldots \ldots \ldots \ldots \ldots ..\\ \left(\lambda_{n1}^{+(z)},\lambda_{n1}^{-(z)}),(\lambda_{n2}^{+(z)},\lambda_{n2}^{-(z)})\ldots (\lambda_{nn}^{+(z)},\lambda_{nm}^{-(z)}\right) \end{array}\right]$$

For the sake of simplicity $(\lambda_{ij}^{+(z)},\lambda_{ij}^{-(z)})=\gamma_{ij}^{\left(z\right)}$, hence the above matrix is symbolized as given below

$$(\gamma^{\left(z\right)},\in )=[\gamma_{ij}^{\left(z\right)}]=\left[\begin{array}{l} \left(\gamma_{11}^{\left(z\right)},\gamma_{12}^{\left(z\right)}\ldots (\gamma_{1n}^{\left(z\right)}\right)\\ \left(\gamma_{21}^{\left(z\right)},\gamma_{22}^{\left(z\right)}\ldots (\gamma_{2m}^{\left(z\right)}\right)\\ \ldots \ldots \ldots \ldots \ldots \ldots \ldots \ldots \ldots ..\\ \gamma_{n1}^{\left(z\right)},\gamma_{n2}^{\left(z\right)}\ldots \gamma_{nm}^{+(z)}) \end{array}\right]$$

For each entry $\gamma_{ij}^{\left(z\right)}$ = $\left(\lambda_{ij}^{+(z)},\lambda_{ij}^{-(z)}\right)$, $\lambda_{ij}^{+(z)}\in [0,1]$ denotes the possibility(satisfaction) of deciding on criteria j, and $\lambda_{ij}^{-(z)}\in [-1,0]$ denotes the impossibility (or dissatisfaction) of deciding on criteria j. If $\lambda_{ij}^{+(z)}$ = 1,then the professional *i* shows the maximum satisfaction to criteria(parameter) j and $\lambda_{ij}^{-(z)}$ = -1,then the professional *i* shows the maximum dissatisfaction degree to criteria(parameter) j.

**Step 3: Construct the weighted decision matrix**

Establish the weighted decision matrix $\gamma^{-(z)}=\gamma_{ij}^{-(z)}$, where

$$\gamma_{ij}^{-(z)}=\Omega_{j}w_{i}\gamma_{ij}^{\left(z\right)}$$

$$=(1-(1-\lambda_{ij}^{+(z)}{)}^{w_{i}}{)}^{w_{j}},(\left({\lambda_{ij}}^{-(z)}{)}^{w_{i}}{)}^{\Omega_{j}}\right)),$$ (3.9)

$$=(\bar{\lambda_{ij}^{+(z)}},{\bar{\lambda_{ij}}}^{\left(z\right)})$$

Here, *w*_*i*_ used as a weight vector for its professional *Ω*_*j*_ is the weight vector for j^th^ parameters and $\gamma^{-(z)}=({\bar{\gamma }}^{\left(z\right)}{)}_{n\times m},{\bar{\gamma }}^{\left(z\right)}$ is known as weighted decision matrices. in this weighted decision matrix, the weights are computed by using the above formula mentioned in Eq (3.9)

**Step 4: Compute the correlation matrix**

The correlation between each value of ${\bar{\gamma }}_{ij}^{\left(z\right)}$ and positive ideal *r*^+^ = (1,0) is obtained as:

$$\rho ({\bar{\gamma_{ij}}}^{\left(z\right)},r^{+})=\frac{C_{BFSS}({\bar{\gamma }}_{ij}^{\left(z\right)},r^{+})}{\sqrt{I_{BFSS}({\bar{\gamma }}_{ij}^{\left(z\right)}).I_{BFSS}(r^{+})}}$$ (3.10)

$$\mathrm{Or}\;\rho_{\begin{array}{c} 2 \end{array}}({\bar{\gamma_{ij}}}^{\left(z\right)},r^{+})=\frac{C_{BFSS}({\bar{\gamma }}_{ij}^{\left(z\right)},r^{+})}{\max\{I_{BFSS}({\bar{\gamma }}_{ij}^{\left(z\right)}).I_{BFSS}(r^{+})\}}$$

By using these formula’s, we get a correlation coefficient matrix which is represented by $\psi^{\left(z\right)}=(\psi_{ij}^{\left(z\right)}{)}_{n\times m}$, (z = 1, 2, 3…k) here $\left(\psi_{ij}^{\left(z\right)}\right)$ is correlation coefficient of each value of weighted decision matrix with the positive ideal *r*^+^.

**Step 5: Computation of positive ideal alternative (PIA) and negative ideal alternative (NIA)**

From the correlation coefficient matrix, we find the indices *h*_*ij*_ and *g*_*ij*_ for each professional *u*_*i*_ and parameter *e*_*j*_ which is $h_{ij}={\mathrm{argmax}}_{\left(z\right)}\{\psi_{ij}^{\left(z\right)}\}$ and $h_{ij}={\mathrm{argmin}}_{\left(z\right)}\{\psi_{ij}^{\left(z\right)}\}$ based on these indices, we find PIA(*L*^+^) and NIA (*L*^−^) as

$$L^{+}=({\bar{\lambda^{+}}}_{ij}^{\left(h_{ij}\right)},{\bar{\lambda^{-}}}_{ij}^{\left(h_{ij}\right)}=[(\lambda^{+}{)}^{+},(\lambda^{-}{)}^{+}{]}_{n\times m}$$ (3.11)

$$L^{-}=({\bar{\lambda^{+}}}_{ij}^{\left(g_{ij}\right)},{\bar{\lambda^{-}}}_{ij}^{\left(g_{ij}\right)}=[(\lambda^{+}{)}^{-},(\lambda^{-}{)}^{-}{]}_{n\times m}$$ (3.12)

**Step 6: Correlation coefficient with PIA**

By using either of proposed correlation coefficient *ρ*_1_ and *ρ*_2_, we compute the correlation coefficient between weighted decision matrix ${\bar{\gamma }}^{\left(z\right)},z=(1,2,3\ldots k)$ , of each alternative *γ*^(*z*)^, (*z* = 1,2…*k*) and PIA(*L*^+^).

P(z)=ρ1(γ¯(z),L+)=CBFSS(γ¯(z),L+)IBFSS(γ¯(z)).IBFSS(L+)

$$={Ρ}^{\left(z\right)}=\rho_{2}({\bar{\gamma }}^{\left(z\right)},L^{+})=\frac{C_{BFSS}({\bar{\gamma }}^{\left(z\right)},L^{+})}{\max[I_{BFSS}({\bar{\gamma }}^{\left(z\right)}).I_{BFSS}(L^{+})}$$ (3.13)

$$=\frac{\sum_{j=1}^{m}\sum_{i=1}^{n}({\bar{\lambda }}_{ij}^{+(z)}.(\lambda^{+}{)}^{+}+{\bar{\lambda }}_{ij}^{-(z)}.(\lambda^{-}{)}^{+}}{\max[\sum_{j=1}^{m}\sum_{i=1}^{n}(({\bar{\lambda }}_{ij}^{+(z)}{)}^{2}+({\bar{\lambda }}_{ij}^{-(z)}{)}^{2}).(\sum_{j=1}^{m}\sum_{i=1}^{n}((\lambda_{ij}^{+}){)}^{+}{)}^{2}+((\lambda^{-}{)}^{+}{)}^{2}}$$ (3.14)

**Step 7: Correlation coefficient with NIA**

Find correlation coefficient between weighted decision matrix ${\bar{\gamma }}^{\left(z\right)}$ (z = 1,2…k) and the NIA(*L*^−^) by using following formulas:

q(z)=ρ1(γ¯(z),L−)=CBFSS(γ¯(z),L−)IBFSS(γ¯(z)).IBFSS(L−)

∑j=1m∑i=1n(λ¯ij+(z).(λ+)−+λ¯ij−(z).(λ−)−∑j=1m∑i=1n((λ¯ij+(z))2+(λ¯ij−(z))2∑j=1m∑i=1n((λ+))−)2+((λ−))−)2 (3.15)

$$q^{\left(z\right)}=\rho_{1}({\bar{\gamma }}^{\left(z\right)},L^{-})=\frac{C_{BFSS}{\bar{(\gamma }}^{\left(z\right)},L^{-})}{\max\{I_{BFSS}({\bar{\gamma }}^{\left(z\right)}).I_{BFSS}(L^{-})\}}$$

$$=\frac{\sum_{j=1}^{m}\sum_{i=1}^{n}({\bar{\lambda }}_{ij}^{+(z)}.(\lambda^{+}{)}^{+}+{\bar{\lambda }}_{ij}^{-(z)}.(\lambda^{-}{)}^{+}}{\max\{\sum_{j=1}^{m}\sum_{i=1}^{n}(({\bar{\lambda }}_{ij}^{+(z)}{)}^{2}+({\bar{\lambda }}_{ij}^{-(z)}{)}^{2}.(\sum_{j=1}^{m}\sum_{i=1}^{n}(({\bar{\lambda }}_{ij}^{+}){)}^{-}{)}^{2}+(((\lambda^{-}){)}^{-}{)}^{2}\}}$$ (3.16)

**Step 8: Computation of closeness coefficient**

The closeness coefficient for each alternative *γ*^(*z*)^, (*z* = 1,2…*k*) is obtained as:

$$R^{\left(z\right)}=\frac{\eta ({\bar{\gamma }}^{\left(z\right)},L^{-})}{\eta ({\bar{\gamma }}^{\left(z\right)},L^{+})+\eta ({\bar{\gamma }}^{\left(z\right)},L^{-})}$$ (3.17)

where $\eta ({\bar{\gamma }}^{\left(z\right)},L^{-})=1-q^{\left(z\right)}$ and $\eta ({\bar{\gamma }}^{\left(z\right)},L^{-})=1-P^{\left(z\right)}$

**Step 9: Ranking the preference order**

By arranging the value of *R*^(*z*)^ in descending order, we give ranks to alternatives and find the best alternative.

In this section below, we describe the application of the proposed technique by giving an example

## 4. Practical Application of Proposed Technique

### 4.1 Diagnosis of specific skin disorder

**Fig 2:**
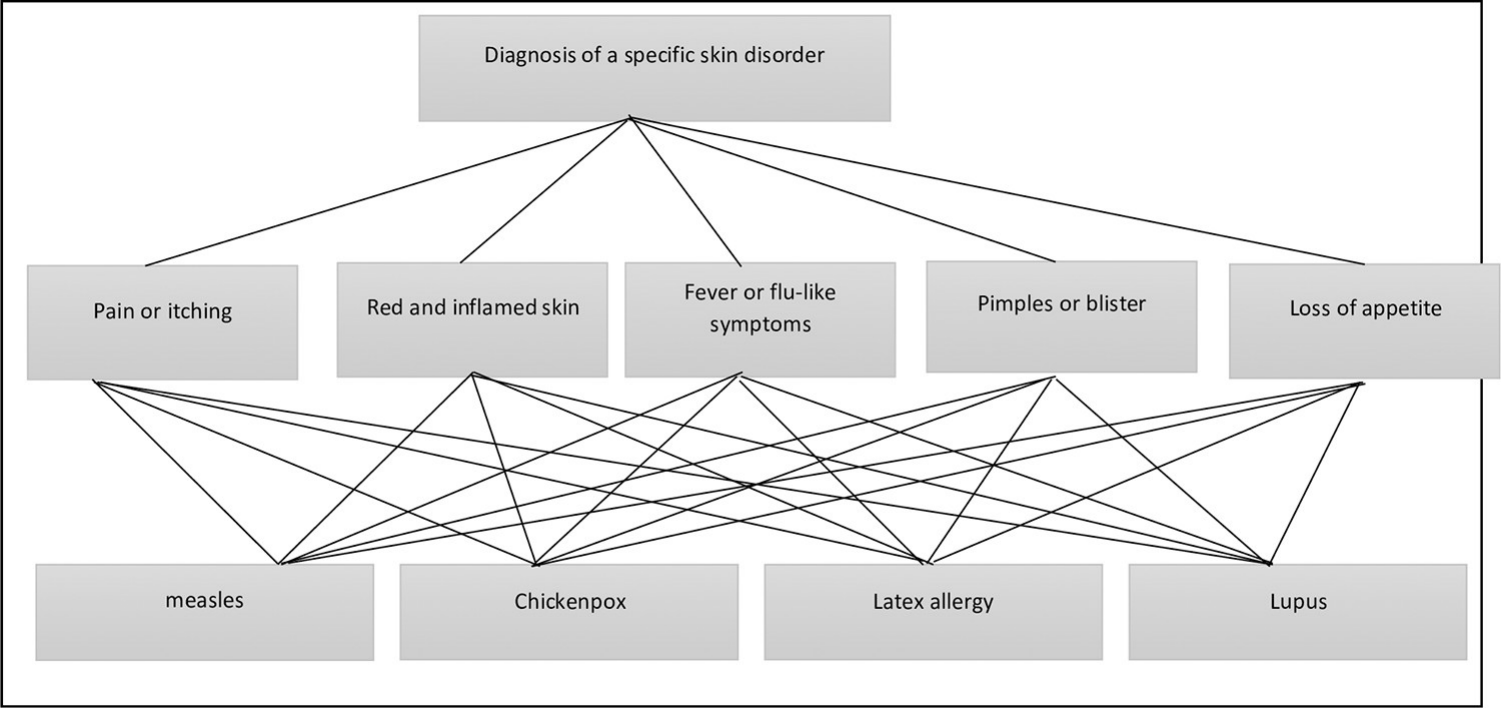
The framework for the diagnosis of a skin disorder.

Medical diagnosis is a procedure in which the occurrence of the disease is found based on the patient’s symptoms. For proper diagnosis lab test as well as physical examination is required. The initial diagnosis starts with signs, symptoms, and later tests. Many health care units evaluate the patient’s health by the domain expert based on signs, symptoms, and investigation. Diagnosis has been made on the basis of the intensity of these signs and symptoms. Assuming that the skincare experts have to select the specific skin disorder by evaluating the symptoms related to five different diseases that include *A*^(1)^ = Measles, *A*^(2)^ = Chicken Pox, *A*^(3)^ = Latex allergy, *A*^(4)^ = Lupus. A group of three decision-makers (professionals) having weights vector (0.5, 0.4, 0.1) decided upon to evaluate these four diseases (alternatives) on the basics of five symptoms, such as *S*_1_ = Pain or itching *S*_2_ = Red and inflamed skin, *S*_3_ = Fever or flu-like symptoms, *S*_4_ = Pimples or blisters, *S*_5_ = loss of appetite. Symptoms are considered as criteria having weights (0.30, 0.15, 0.25, 0.20, 0.1). These weights are assigned by the professionals (expert doctors) after specifying the intensity of signs or symptoms for a particular patient. This intensity was rated as mild, moderate, severe, very severe. The framework for evaluating the diagnosis strategy is provided in Fig 2

Here the objective is to find out the specific disease on the basis of criteria (symptoms) mentioned by the decision-makers.

**Step 1:** The professional (Doctors**) ratings** for each symptom are summarized in Table (1–4).

**Table 1:** Alternative *A*^(1)^.

*A* ^(1)^	*****S**_**1**_***	*****S**_**2**_***	*****S**_**3**_***	*****S**_**4**_***	*****S**_**5**_***
***D_1_***	(0.5,-0.1)	(0.7,-0.3)	(0.9,-0.1)	(0.6,-0.2)	(0.7,-0.2)
***D_2_***	(0.6,-0.2)	(0.5,-0.4)	(0.8,-0.3)	(0.5,-0.4)	(0.8,-0.1)
***D_3_***	(0.5,-0.4)	(0.8,-0.2)	(0.6,-0.3)	(0.5,-0.1)	(0.7,-0.1)

**Table 2:** Alternative *A*^(2)^.

*A* ^(2)^	*****S**_**1**_***	*****S**_**2**_***	*****S**_**3**_***	*****S**_**4**_***	*****S**_**5**_***
***D_1_***	(0.9,-0.1)	(0.8,-0.2)	(0.9,-0.1)	(0.7,-0.3)	(0.6,-0.5)
***D_2_***	(0.7,-0.3)	(0.9,-0.1)	(0.7,-0.3)	(0.9,-0.1)	(0.7,-0.3)
***D_3_***	(0.6,-0.1)	(0.7,-0.1)	(0.5,-0.2)	(0.8,-0.2)	(0.6,-0.4)

**Table 3:** Alternative *A*^(3)^.

*A* ^(3)^	*****S**_**1**_***	*****S**_**2**_***	*****S**_**3**_***	*****S**_**4**_***	*****S**_**5**_***
***D_1_***	(0.4,-0.3)	(0.9,-0.1)	(0.8,-0.1)	(0.4,-0.6)	(0.5,-0.6)
***D_2_***	(0.5,-0.4)	(0.6,-0.4)	(0.7,-0.2)	(0.5,-0.5)	(0.3,-0.7)
***D_3_***	(0.6,-0.5)	(0.5,-0.5)	(0.8,-0.2)	(0.4,-0.7)	(0.2,-0.8)

**Table 4:** Alternative *A*^(4)^.

*A* ^(4)^	*****S**_**1**_***	*****S**_**2**_***	*****S**_**3**_***	*****S**_**4**_***	*****S**_**5**_***
***D_1_***	(0.9,-0.1)	(0.7,-0.2)	(0.7,-0.4)	(0.1,-0.9)	(0.2,-0.9)
***D_2_***	(0.8,-0.2)	(0.7,-0.1)	(0.6,-0.3)	(0.2,-0.6)	(0.1,-0.9)
***D_3_***	(0.7,-0.1)	(0.9,-0.1)	(0.8,-0.3)	(0.1,-0.7)	(0.3,-0.6)

Table (1– 4) shows the bipolar fuzzy decision matrix, where each entry corresponds to each decision-maker, and particular symptom represents the positive and negative membership values. The positive membership degree shows the possibility of alternative under specific characteristics, and the negative membership degree shows the impossibility of alternative under specific characteristics. Suppose the value of positive membership degree ($\lambda_{ij}^{+}$) is close to 1. In that case, the alternative represents the maximum possibility of occurrence to criteria. If the value of negative membership degree *λ*_*ij*_^−^ is close to -1, then the alternative represents the maximum impossibility of occurrence to criteria.

**Step 2:** Compute the weighted decision matrix *γ*^−(*z*)^ where (z= 1, 2, 3, 4) by using Eq (3.9). The results obtained are summarized in Tables (5–8).

**Table 5:** Weighted bipolar fuzzy decision matrix *γ*^(1)^.

	*****S**_**1**_***	*****S**_**2**_***	*****S**_**3**_***	*****S**_**4**_***	*****S**_**5**_***
***D_1_***	(0.0798,- 0.7585)	(0.0697,- 0.9303)	(0.2057, -0.7943)	(0.9600,- 0.8792)	(0.0470,- 0.9377)
***D_2_***	(0.1284,- 0.8347)	(0.0507,- 0.9336)	(0.1822, -0.8603)	(0.9330,- 0.9124)	(0.0773,- 0.8913)
***D_3_***	(0.0206,- 0.9332)	(0.0239,- 0.9761)	(0.0226,- 0.9703)	(0.9862, -0.9550)	(0.0120,- 0.9772)

**Table 6:** Weighted bipolar fuzzy decision matrix *γ*^(2)^.

	*****S**_**1**_***	*****S**_**2**_***	*****S**_**3**_***	*****S**_**4**_***	*****S**_**5**_***
***D_1_***	(0.2414,- 0.7586)	(0.0921,- 0.9079)	(0.2057,- 0.7943)	(0.0918,- 0.9082)	(0.0360,- 0.9727)
***D_2_***	(0.1652,- 0.8348)	(0.1586,-0.8414)	(0.1397,- 0.8603)	(0.2057,- 0.7943)	(0.0584,- 0.9416)
***D_3_***	(0.0271, -0.9333)	(0.0921,- 0.9661)	(0.0172,- 0.9606)	(0.0317,- 0.9683)	(0.0091,- 0.9909)

**Table 7:** Weighted bipolar fuzzy decision matrix *γ*^(3)^.

	*****S**_**1**_***	*****S**_**2**_***	*****S**_**3**_***	*****S**_**4**_***	*****S**_**5**_***
***D_1_***	(0.0595, -0.8655)	(0.1290,- 0.8710)	(0.1487,- 0.7943)	(0.0400,- 0.9600)	(0.0273,- 0.9798)
***D_2_***	(0.0987,- 0.8716)	(0.0664,- 0.9336)	(0.1397,- 0.8178)	(0.0670,- 0.9330)	(0.0177,- 0.9823)
***D_3_***	(0.0271, -0.9794)	(0.0103,- 0.9897)	(0.0394,- 0.9606)	(0.0238,- 0.9929)	(0.0022,- 0.9978)

**Table 8:** Weighted bipolar fuzzy decision matrix *γ*^(4)^.

	*****S**_**1**_***	*****S**_**2**_***	*****S**_**3**_***	*****S**_**4**_***	*****S**_**5**_***
***D_1_***	(0.2414,- 0.7586)	(0.0697,- 0.9079)	(0.1134,- 0.9124)	(0.0084,- 0.9916)	(0.0089,- 0.9958)
***D_2_***	(0.2145,- 0.7855)	(0.0863,- 0.8414)	(0.1082, -0.8603)	(0.0221,- 0.9502)	(0.0053,- 0.9947)
***D_3_***	(0.0355,- 0.9333)	(0.0339,- 0.9661)	(0.0394, -0.9703)	(0.0021,- 0.9929)	(0.0036,- 0.9949)

The above Tables (5–8) represents the weighted bipolar fuzzy decision matrix. This matrix is obtained by multiplying the bipolar decision matrix to weight vector for *i*th professional and weight vector for the parameter.

**Step 3:** Compute the correlation coefficients between each alternative *γ*^(*z*)^, (*z* = 1,2,3,4) and positive ideal *r*^+^ by utilizing Eq (3.10)

$$\psi^{\left(1\right)}=[\begin{array}{lllll} 0.9257 & 0.7581 & 0.8618 & 0.9990 & 0.7416\\ 0.8064 & 0.7443 & 0.8383 & 0.9999 & 0.7656\\ 0.7225 & 0.7225 & 0.9704 & 0.9999 & 0.7157 \end{array}]$$

$$\psi^{\left(2\right)}=[\begin{array}{lllll} 0.8882 & 0.8326 & 0.8618 & 0.7746 & 0.7327\\ 0.8309 & 0.7710 & 0.8113 & 0.8618 & 0.7495\\ 0.7273 & 0.8618 & 0.7197 & 0.7299 & 0.7136 \end{array}]$$

$$\psi^{\left(3\right)}=[\begin{array}{lllll} 0.7539 & 0.8032 & 0.8251 & 0.7359 & 0.7266\\ 0.7822 & 0.7555 & 0.8161 & 0.7559 & 0.7197\\ 0.7264 & 0.7144 & 0.7355 & 0.7238 & 0.7087 \end{array}]$$

$$\psi^{\left(4\right)}=[\begin{array}{lllll} 0.8882 & 0.7592 & 0.7829 & 0.7131 & 0.7134\\ 0.8684 & 0.7756 & 0.7898 & 0.7234 & 0.7109\\ 0.7335 & 0.7315 & 0.7352 & 0.7086 & 0.7116 \end{array}]$$

The above matrix from ψ^(1)^ *to* ψ^(2)^ represents the correlation coefficient matrix, in which each value shows the correlation coefficient. This correlation coefficient is computed for each value of the weighted decision matrix and perfect ideal r+ by applying the Eq (3.9).

Step 4: Compute the PIA and NIA by using Eqs (3.11) and (3.12)

$$L^{+}=[\begin{array}{lllll} \left(0.2414,-0.8655\right) & \left(0.1290,-0.9303\right) & \left(0.2057,-0.9124\right) & \left(0.9600,-0.9916\right) & \left(0.0470,-0.9958\right)\\ \left(0.2145,-0.8716\right) & \left(0.1586,-0.9336\right) & \left(0.1822,-0.8603\right) & \left(0.9330,-0.9502\right) & \left(0.0773,-0.9947\right)\\ \left(0.0355,-0.9794\right) & \left(0.0921,-0.9897\right) & \left(0.0394,-0.9703\right) & \left(0.9862,-0.9929\right) & \left(0.0120,-0.9949\right) \end{array}]$$

$$L^{-}=[\begin{array}{lllll} \left(0.0595,-0.7585\right) & \left(0.0697,-0.8710\right) & \left(0.1134,-0.7943\right) & \left(0.0084,-0.8792\right) & \left(0.0089,-0.9377\right)\\ \left(0.0988,-0.8347\right) & \left(0.0507,-0.8414\right) & \left(0.1082,-0.8178\right) & \left(0.0221,-0.7943\right) & \left(0.0053,-0.0053\right)\\ \left(0.0206,-0.9332\right) & \left(0.0103,-0.9661\right) & \left(0.0172,-0.9606\right) & \left(0.0021,-0.9550\right) & \left(0.0022,-0.9772\right) \end{array}]$$

The above Table shows the PIA and NIA, and these ideals are computed on the basis of the indices (*h*_*ij*_ and *g*_*ij*_), where *h*_*ij*_ = *argmax*_(*z*)_ {*ψ*_*ij*_^(*z*)^} and *g*_*ij*_ = *argmin*_(*z*)_ {*ψ*_*ij*_^(*z*)^}.

**Step 5:** Correlation coefficient between each alternative of the weighted decision matrix ${\bar{\gamma }}^{\left(z\right)}$ and PIA *L*^+^ is computed by using Eq (3.13) and, *ρ*^(1)^ = 0.9268, *ρ*^(2)^ = 0.9268, *ρ*^(3)^ = 0.9083, *ρ*^(4)^ = 0.8984 are obtained.

The above values represents the interrelationship between each alternative and positive ideal.

**Step 6:** Correlation coefficient between each alternative of the weighted decision matrix ${\overset{-}{\gamma }}^{\left(z\right)}$ and NIA ℒ^−^ is computed by using Eq (3.15) and *q*^(1)^ = 0.8715, *q*^(2)^ = 0.9999, *q*^(3)^ = 0.9518, *q*^(4)^ = 0.9349 are obtained.

The association between each alternative and negative ideal is mentioned above.

**Step 7:** By using Eq (3.17), we get the closeness coefficient of alternatives given as *R*^(1)^ = 0.9421 *R*^(2)^ = 0.5056, *R*^(3)^ = 0.3445, *R*^(4)^ = 0.3905. The higher value of the closeness coefficient represents the best alternative.

**Step 8**: By analyzing the closeness coefficient, we conclude that *R*^(1)^>*R*^(2)^>*R*^(4)^>*R*^(3)^, so the ranking of the alternative is *A*^(1)^>*A*^(2)^>*A*^(4)^>*A*^(3)^. Therefore, *A*^(1)^ is the best alternative.

In the above step, by arranging the closeness coefficient in descending order, we conclude the result that the first closeness coefficient (*R*^(1)^) has a higher value, so alternative one (*A*^(1)^) is the best alternative. As a result, the disease measles is diagnosed. The maximum of which best describes the symptoms mentioned.

## 6. Discussion and comparative study

The following section will discuss the effectiveness, adaptability, and advantages of the suggested technique and algorithm. We also put together a quick comparison of the suggested and existing methodologies.

### 6.1 BF-TOPSIS is used to select the best alternative

To justify the predominance of our methodology, this section comprises the comparative investigation of the proposed approach with the prevailing methodology (Akram et al., 2020). The obtained results so described are as follows:

To implement the BF- TOPSIS approach, we utilize the information as presented in tables (4.5 to 4.8). Based on these tables, we compute weighed bipolar fuzzy decision matrix. After finding BFPIS and BFNIS, the results are as follows.

BFPIS= [(0.24, -0.06), (0.12, -0.015), (0.2, -0.05), (0.16, -0.04), (0.07, -0.01)]

BFNIS= [(0.15, -0.12), (0.105, -0.045), (0.175, -0.08), (0.08, -0.14), (0.02, -0.08)]

Euclidean distance of each disease from BFPIS and BFNIS is given as

$$d(A^{\left(1\right)},BFPIS)=0.1007\;d(A^{\left(1\right)},BFNIS)=0.07793$$

$$d(A^{\left(2\right)},BFPIS)=0.0024\;d(A^{\left(2\right)},BFNIS)=0.00677$$

$$d(A^{\left(3\right)},BFPIS)=0.0159\;d(A^{\left(3\right)},BFNIS)=0.00138$$

$$d(A^{\left(4\right)},BFPIS)=0.1473\;d(A^{\left(4\right)},BFNIS)=0.00086$$

and relative closeness coefficient of the given diseases is obtained as

*R*^(1)^ = 0.4364, *R*^(2)^ = 0.7391, *R*^(3)^ = 0.0809, *R*^(4)^ = 0.0061.

**Fig 3:**
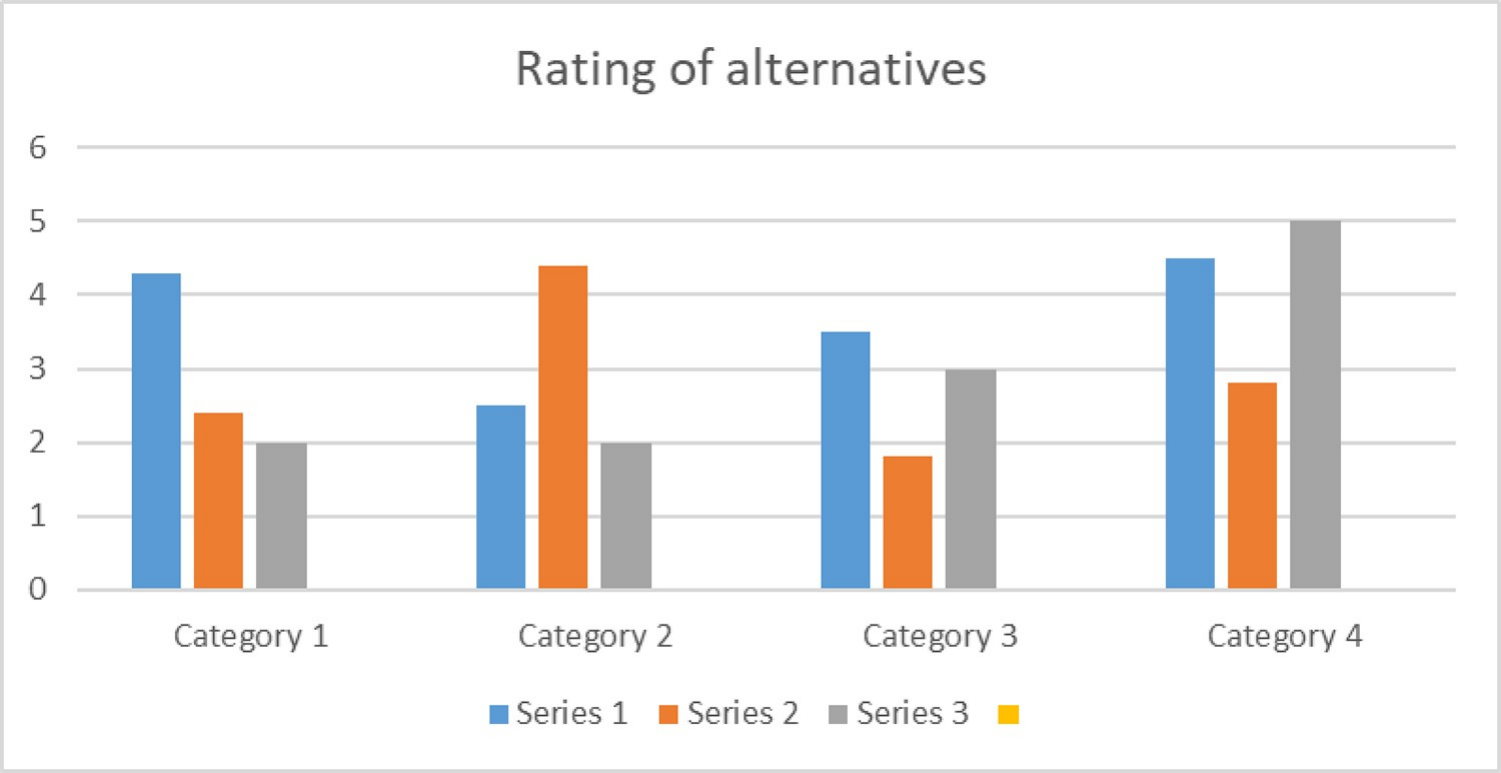
Ranking of alternatives by using BF-TOPSIS and proposed technique.

Hence, the ranking order is *A*^(2)^>*A*^(1)^>*A*^(3)^>*A*^(4)^. Therefore, *A*^(2)^ is the best alternative, the same as that of the proposed approach, but the values of closeness coefficient in our proposed method are close to 1, which shows more preciseness in the result. The above description is represented in Fig 3.

We deal with accurate information of the alternatives using the methodology of (Zadeh 1965), although this method cannot deal with falsity objects or many sub-attributes of the alternatives. In [29], H. M. Zhang et al., 2007) are unable to present dual features of the problem and many sub-attributes of the alternatives. It has been mentioned that the (Garg & Arora, 2020), see [25] and (Rana Muhammad Zulqarnain et al., 2021), [28] Methodologies are unable to deal with the dual aspect information’s. Our proposed technique may readily overcome these challenges and give more effective outcomes for MADM difficulties. Instead, our proposed method is a sophisticated strategy that can deal with multiple sub-attribute information alternatives. The Table 9 as mentioned above shows a comparison and figure 9 presents the comparison of bipolar fuzzy Topsis and the proposed technique. On the other hand, the methodology, we developed addresses the dual issues of alternatives with sub-attribute and their inaccuracy. As compared to existing methodologies, the technique we developed is more efficient and can deliver successful results for decision-makers using an array of perspectives.

**Table 9:** Analysis of existing procedures in comparison to the proposed method.

	Set	Dual-aspects	Attributes	Sub-Attributes	Falsity	Loss of data
Zadeh	FS	×	√	×	×	√
Zhang et al.	IFS	×	√	×	√	√
Greg &Arora	IFSS	×	√	√	√	√
Zulqarnain et al.	IVFSS	×	√	×	√	×
Proposed approach	BFSS	√	√	√	√	×

## Conclusion

The investigated research manipulates the BFSSs to deal with the unsatisfactory and incompatible data by assuming the possibility and impossibility degrees over the set of parameters. The basic intuition of the correlation coefficient and weighted correlation coefficient for BFSSs with their properties is formulated in this research. A new algorithm has been introduced on the basics of the correlation coefficient by taking into account the set of attributes and experts. The suggested method could be helpful in future for medical diagnosis in dual or bipolar behavior. The correlation matrix has been formulated by using correlation indices. PIA and NIA are also determined. To locate the hierarchy of the alternative, we defined the closeness coefficient for the proposed technique. Finally, a numerical illustration of the diagnosis has been described. The following Table 10 represents the comparison of the existing and proposed techniques as described in Table 9.

**Table 10:** Comparison of the existing and proposed techniques.

Existing Method	Proposed technique
The existing method neglects the preference information of decision-makers. Hence, it does not provide precise results.	Using soft sets in our proposed technique, we consider the preference information, leading to a more compressed and precise result.
PIA and NIA are computed by taking the extreme values based on the given decision matrix.	The computational procedure is different for the determination of ideal solutions. PIA and NIA are determined on the given alternative rating based on the impact of the maximum correlation coefficient. This leads to less loss of information during the process.
This method considers the only degree of similarity between the observations.	The correlation measures take into account the degree of similarity and the degree of discrimination between the observations. This helps avoid the decision which is only based on negative reasons.
The utilization of Euclidean distance does not think about the relationship of attributes.	Our proposed method is based on correlation coefficient; that’s why it effectively bothers the relationship of attributes and alternatives.
It is insufficient to only consider the evaluation information from individual decision-makers when making the decision results.	Our proposed method overcome this insufficiency by considering a group of three decision-maker for the evaluation of information.

## Supporting information

S1 Checklist PRISMA-P (Preferred Reporting Items for Systematic review and Meta-Analysis Protocols) 2015 checklist: Recommended items are addressed in a systematic review protocol*. (DOC)

S1 File (DOC)
