# Supplementary material for: An application of fuzzy bipolar weighted correlation coefficient in decision-making problem
Source: PLoS One. 2023 Dec 19;18(12):e0283516. doi: 10.1371/journal.pone.0283516 (PMC10729986; doi:10.1371/journal.pone.0283516)
Supplement: S1 File — (DOC) [file pone.0283516.s002.doc]

**Data Information**

There following tables shows the rating of alternatives in terms of linguistic scale, where there are four alternatives and three decision makers for the specification of a particular diseases on the bases of symptoms.

The tables (1 - 4) below represent the decisions made by decision maker in four linguistic terms where, Mild represented by (ML), Moderate by(M), Severe represented by (S) and Very Severe (VS).

**Table 1:** Rating for Alternative 1 in terms of linguistic scale

| Symptoms/criteria | DM1 | DM2 | DM3 |
| --- | --- | --- | --- |
| 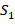 | M | M | M |
| 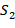 | S | M | S |
| 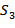 | VS | S | S |
| 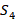 | M | M | M |
| 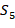 | S | VS | S |

**Table 2:** Rating for Alternative 2 in terms of linguistic scale

| Symptoms/criteria | DM1 | DM2 | DM3 |
| --- | --- | --- | --- |
| 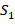 | VS | S | S |
| 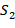 | VS | VS | S |
| 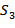 | VS | S | M |
| 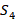 | S | VS | S |
| 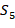 | M | S | M |

**Table 3:** Rating for Alternative 3 in terms of linguistic scale

| Symptoms/criteria | DM1 | DM2 | DM3 |
| --- | --- | --- | --- |
| 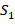 | ML | M | M |
| 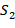 | VS | M | M |
| 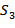 | VS | S | VS |
| 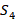 | ML | M | ML |
| 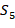 | M | ML | ML |

**Table 4:** Rating for Alternative 4 in terms of linguistic scale

| Symptoms/criteria | DM1 | DM2 | DM3 |
| --- | --- | --- | --- |
| 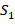 | VS | VS | S |
| 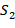 | S | S | VS |
| 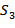 | S | M | VS |
| 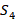 | ML | ML | ML |
| 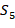 | ML | ML | ML |

| **Calculated Correlation Coefficient Matrix** | | | | | |
| --- | --- | --- | --- | --- | --- |
| ψ1 | 0.9257 | 0.7581 | 0.8618 | 0.999 | 0.7416 |
|  | 0.8064 | 0.7443 | 0.8383 | 0.9999 | 0.7656 |
|  | 0.7225 | 0.7225 | 0.9704 | 0.9999 | 0.7157 |
| ψ2 |  | | | | |
|  | 0.8882 | 0.83258 | 0.8618 | 0.7746 | 0.7327 |
|  | 0.8309 | 0.771 | 0.8113 | 0.8618 | 0.7495 |
|  | 0.7273 | 0.8618 | 0.7197 | 0.7299 | 0.7136 |
| ψ3 |  | | | | |
|  | 0.7539 | 0.8032 | 0.8251 | 0.7359 | 0.7266 |
|  | 0.7822 | 0.7555 | 0.8161 | 0.7559 | 0.7197 |
|  | 0.7264 | 0.7144 | 0.7355 | 0.7238 | 0.7087 |
| ψ4 |  | | | | |
|  | 0.88823 | 0.75916 | 0.78287 | 0.7131 | 0.7134 |
|  | 0.8684 | 0.77556 | 0.78982 | 0.7234 | 0.71086 |
|  | 0.73347 | 0.73147 | 0.73521 | 0.7086 | 0.71157 |

**Competition of Correlation Coefficient with Positive Ideal Alternative**
